# Idiopathic Retroperitoneal Non-pancreatic Pseudocyst in an Adult Male: Radiological Images and Surgical Video of Laparoscopic Excision

**DOI:** 10.7759/cureus.7243

**Published:** 2020-03-11

**Authors:** Ejaz Latif, Shameel Musthafa, Aryan Ahmed, Maneesh Khanna

**Affiliations:** 1 General Surgery, Hamad Medical Corporation, Doha, QAT; 2 Acute Care Surgery, Hamad Medical Corporation, Doha, QAT; 3 Radiology, Wollongong Diagnostics, Wollongong, AUS

**Keywords:** pseudocyst, retroperitoneal cyst, laparoscopic surgery, non-pancreatic

## Abstract

Retroperitoneal pseudocyst (RPC) is an uncommon surgical entity. The pseudocyst is characterized by the absence of epithelial lining in the cyst wall. Mostly, it occurs as a sequela of pancreatitis. Pseudocyst due to a non-pancreatic cause, however, is very rare.

We report a 49-year-old male, who presented to the emergency department with moderate intensity lower abdominal pain. Computed tomography scans revealed a huge retroperitoneal cyst which was overlying the right ureter and right iliac vessels. The patient underwent laparoscopic excision of the cyst and recovered without any complications. Histopathological examination showed a non-pancreatic RPC.

In conclusion, non-pancreatic RPC is a rare surgical disease which can result in pressure symptoms depending on its location and size. In our patient, it was treated by laparoscopic excision despite its proximity to iliac vessels and ureter.

A laparoscopic approach using safe surgical principles is a viable option for non-pancreatic RPC. The proximity of the lesion to the iliac vessels and the ureter can be carefully navigated safely by laparoscopy. The ureter can be confirmed by stimulating peristalsis of the duct when in doubt.

## Introduction

Retroperitoneal cysts are uncommon with a reported incidence of 1 in 5750 to 1 in 250,000 (average 1 in 105,000) [1]. These cysts are lined with epithelium. A retroperitoneal pseudocyst (RPC), on the contrary, is defined as a fluid-filled cavity that lacks an epithelium and is usually lined by fibrous tissue. Commonly, pseudocysts occur as a complication of acute pancreatitis. Primary non-pancreatic RPC or idiopathic RPC occurs due to unknown causes and is rarely reported in the literature [2]. To date, there is no available data published compiling all reported cases due to the rarity of the pathology itself. As retroperitoneum is a potential space composed of loose areolar tissue, such cysts can attain massive size before becoming symptomatic. Here we describe a rare case of non-pancreatic RPC and our laparoscopic surgical approach for the treatment.

## Case presentation

A 49-year-old Indian male with no known co-morbidities, presented with right iliac fossa pain for one day. The pain started gradually and progressively increased in severity. It was non-migratory and non-radiating without any aggravating factors.

The patient has a history of constipation for 5-6 years for which he was taking laxatives intermittently. There was no history of weight loss or bleeding per rectum, similar prior episodes, night sweats nor any contact with tuberculosis patients. His family history was non-contributory. His past medical history was significant for left inguinal hernia repair.

At presentation, his vitals were within normal range. His abdomen was non-tender, non-distended and soft to touch. He had mild fullness in right lower quadrant. However, no definite mass was palpable. Digital rectal exam was normal.

Investigations

His labs revealed WBC of 11,400 per microliters with neutrophil of 82%, hemoglobin was 15 gm/dl, platelets 273,000 per microliters, rest of the labs were within normal limits. Quantiferon test for tuberculosis was negative and Echinococcus antibody titer was normal. Tumor markers carcinoembryonic antigen (CEA) and Alfa fetoprotein (AFP) were done and were normal.

**Figure 1:**
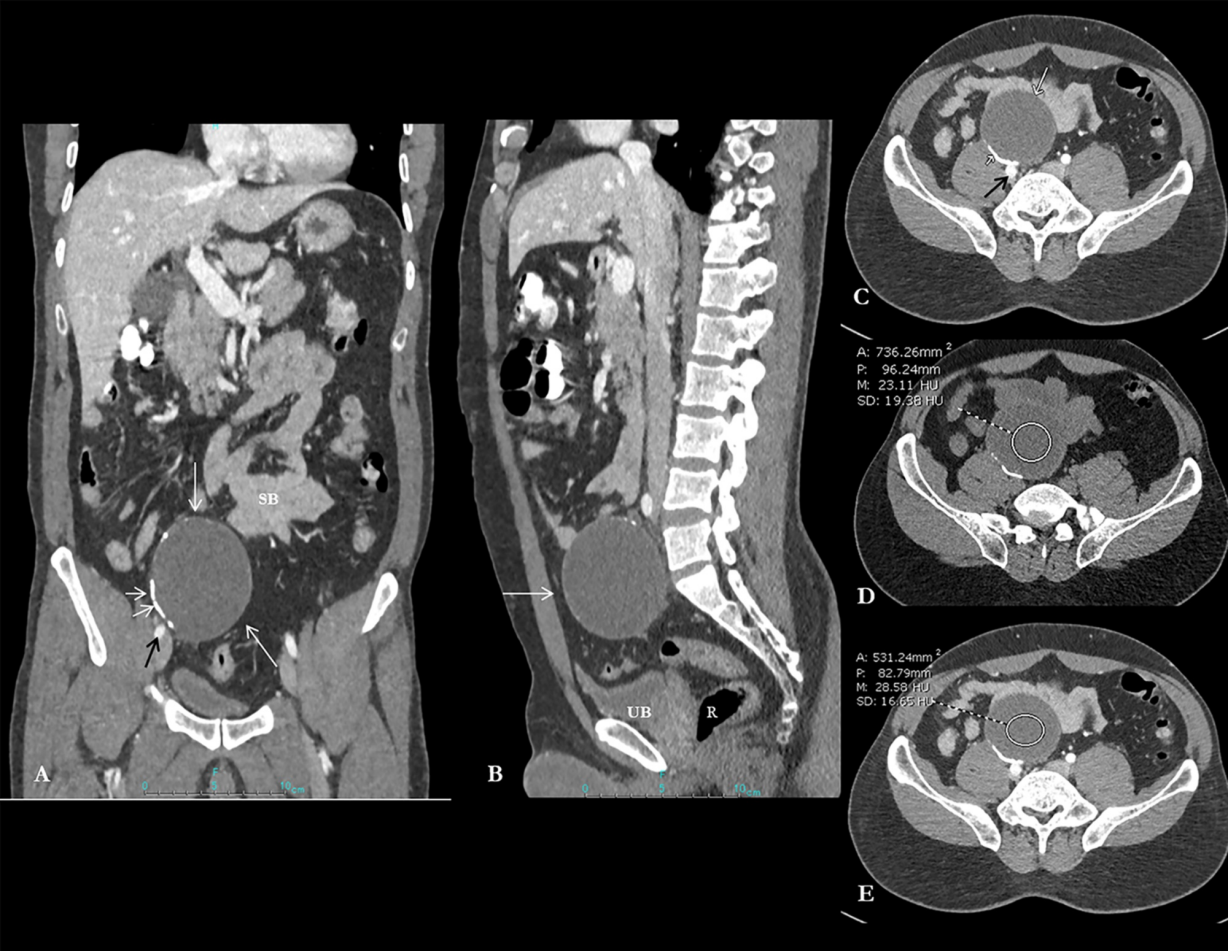
Composite image showing contrast-enhanced CT abdomen and pelvis coronal reformatted views Contrast-enhanced CT abdomen and pelvis coronal reformatted image (A) demonstrates a fairly well-circumscribed oval cystic lesion in the right hemipelvis (long white arrow). The cyst is lying anterior and separate from the right common iliac vessels (black arrow; image A and axial image C), urinary bladder (UB), rectum (R) and small bowel loops (SB) (image A, B and C). Note the presence of curvilinear hyperdense calcification (short white arrows; image A, C). Axial unenhanced image (D) and post-contrast image (E) confirm the cystic unenhanced nature of the lesion (23-28 HU).

The patient underwent CT scan, which revealed a 7 x 6 cm cystic lesion with incomplete peripheral calcification in pelvis more to right side (Figure 1A). The cyst is lying anterior and separate from the right common iliac vessels, urinary bladder, rectum, and small bowel loops (Figure 1A-1C). The lesion appears unenhanced on CT scan (Figure 1D, 1E).

Differential diagnosis

The differentials of this lesion in the right lower quadrant were benign and malignant cystic lesions. We considered it a benign cyst, based on the history of no weight loss, normal tumor markers and radiological findings of a well-defined cyst on CT scan which does not appear to involve or invade any surrounding structures. One of the possibilities was an echinococcal cyst but it was ruled out by the absence of daughter cysts, non-enhancing wall or floating membrane, and normal echinococcal titers. The other differentials were neoplastic cystic lesions but it was less likely as the cystic mass had a well-defined wall without any features of invasion to surrounding structures.

It was really challenging to figure out the diagnosis based on history and imaging. That’s why we opted for laparoscopic excision of the mass to establish a definitive diagnosis.

Treatment

The patient underwent laparoscopic cyst excision (Video 1).

**Video 1:** Laparoscopic excision of retroperitoneal pseudocyst Intra-operative video of the laparoscopic surgery showing dissection in all directions around the mass and its relation to sigmoid, iliac vessels, and right ureter. All vital structures are tagged in the video as well as voice over to explain the steps of the procedure. Stimulation of the ureter to induce peristalsis is demonstrated at 3 min, 55 sec which helped in identifying it.

The location of the cyst was clearly the retroperitoneum as there was a complete peritoneal covering over the lesion. The cyst was compressing the right ureter and in proximity with the right internal iliac vessels. It was dissected carefully from the right ureter and right iliac vessels using Harmonic scalpel and safe laparoscopic surgical principles. Identification of the ureter by gently stimulating peristalsis is clearly demonstrated in the video. Inadvertent opening of the cyst occurred during dissection of the posteromedial part of the cyst due to a very thin false capsule. The fluid content of the cyst was aspirated and tested for amylase and tumor markers (AFP and CEA) but was not detected, confirming the diagnosis of a non-pancreatic pseudocyst. The mass was excised completely and removed using a specimen retrieval bag after copious irrigation and ensuring good hemostasis.

Outcome & follow-up

Postoperatively, the patient recovered well with no complications. Oral fluids were started on postoperative day 1 which was progressed to full diet on the same day. On postoperative day 2, the patient was pain-free, had passed stool, had adequate urine output and was discharged home. After two weeks, he was followed up in the clinic with a normal abdominal examination and well-healing wounds.

**Figure 2:**
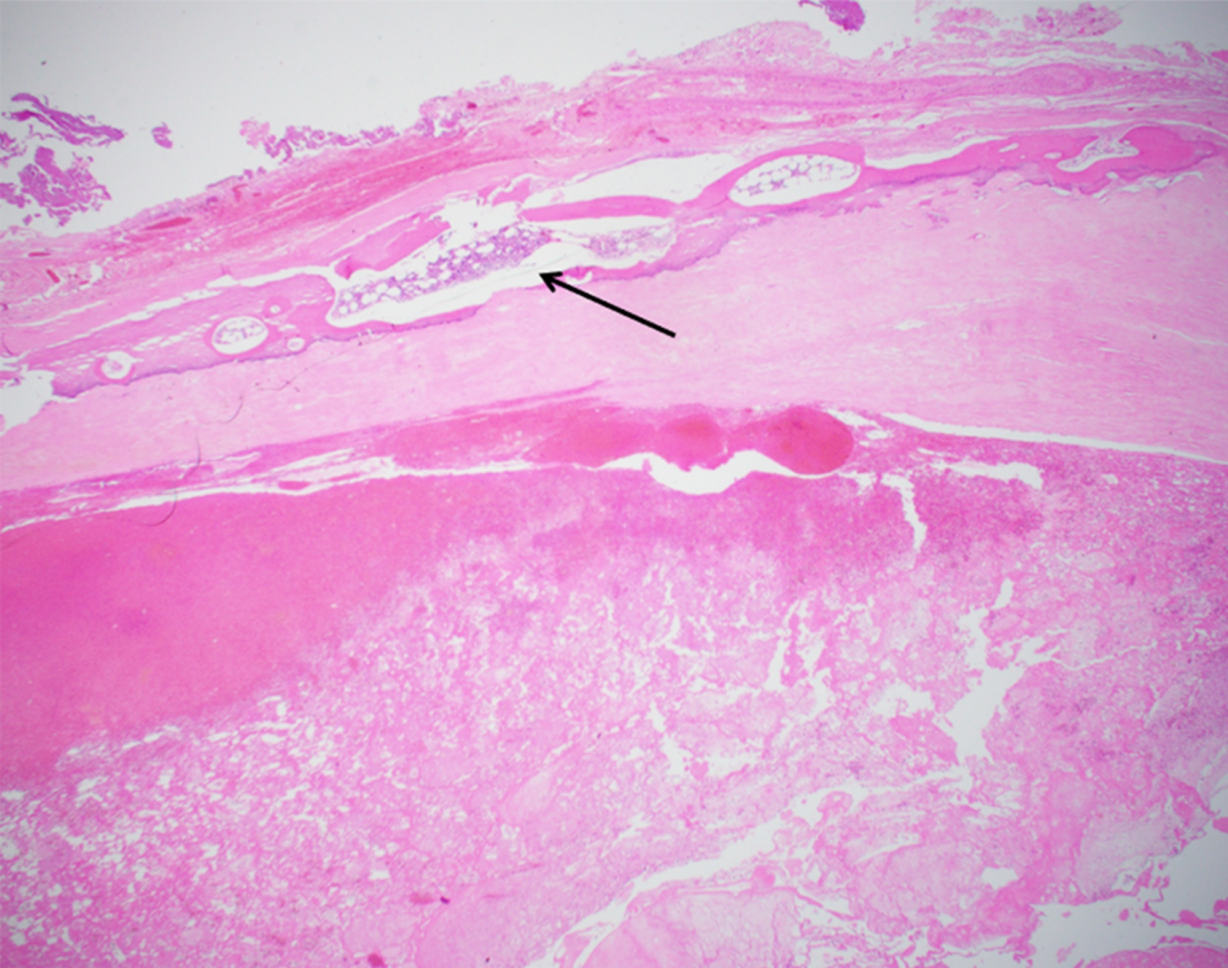
Photomicrograph, low-power view (Hematoxylin and Eosin, x20) Low-power view shows cyst wall composed of fibrous tissue with hematopoietic elements (black arrow).

**Figure 3:**
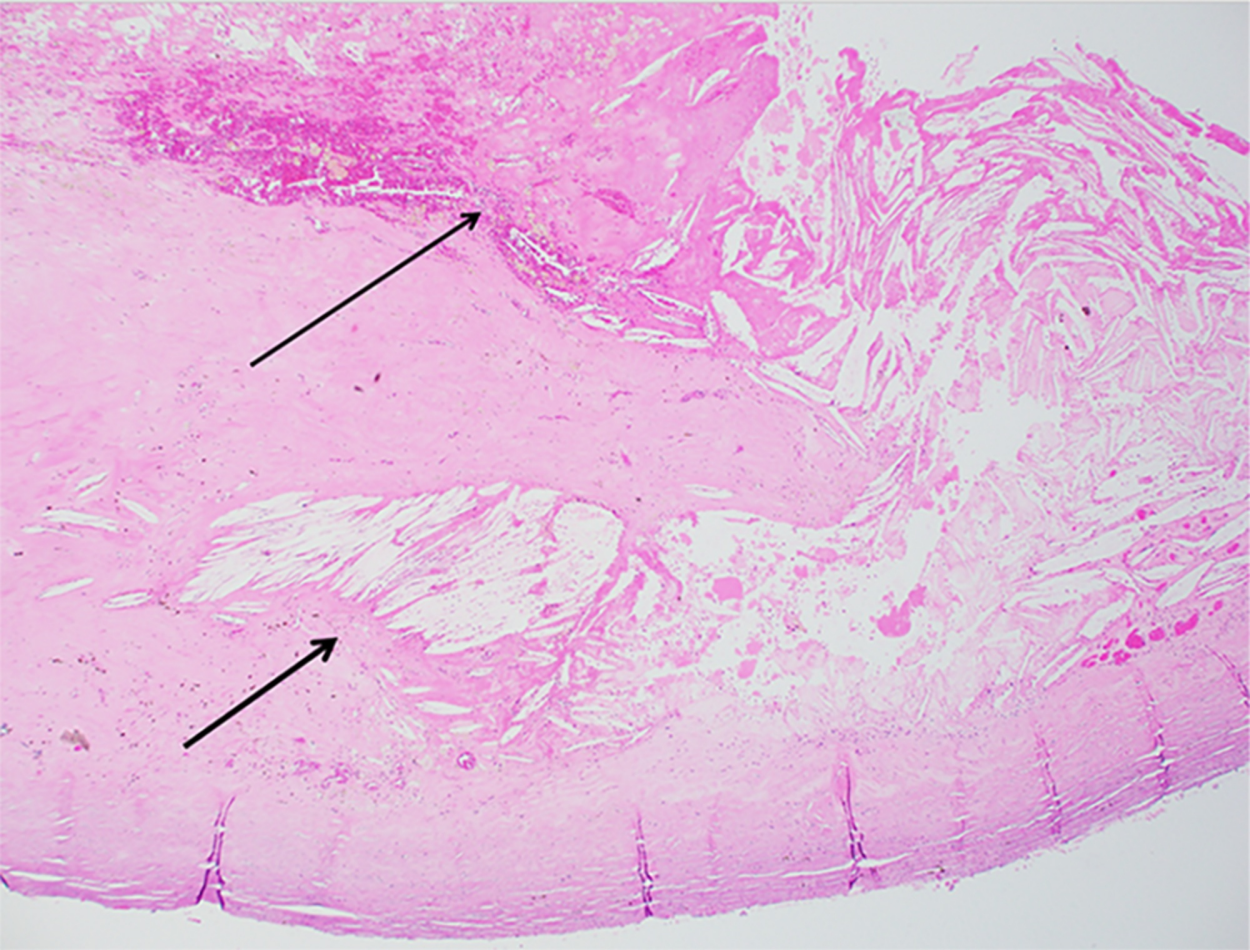
Photomicrograph, low-power view (Hematoxylin and Eosin, x20) Photomicrograph showing cyst wall with cholesterol clefts (Short black arrow) and fibrin (Long black arrow). Note the absence of epithelial lining.

His histopathology showed a benign fibrous cyst wall containing fibrin with cholesterol clefts. There was no epithelial or endothelial lining and it was negative for granulomas or malignancy (Figure 2 and Figure 3).

## Discussion

Etiology

Retroperitoneal cysts were described by Handfield in 1929, as cysts that arise in the fatty tissue in the retroperitoneum which have no connection to adult anatomical structure except the areolar tissue [3].

Yang et al. classified these cysts into neoplastic and non-neoplastic lesions [4]. Neoplastic lesions are cystic lymphangioma, mucinous cystadenoma, cystic teratoma, cystic mesothelioma, Mullerian cyst, epidermoid cyst, tailgut cyst, bronchogenic cyst, pseudomyxoma retroperitonei, cystic change in solid neoplasms, and perianal mucinous carcinoma. Non-neoplastic lesions include pancreatic pseudocyst, non-pancreatic pseudocyst, hematoma, lymphocele, and urinoma.

Although RPCs are classified generally as retroperitoneal cysts, one should consider that they lack a true epithelium and are, therefore, considered a separate entity. Pseudocysts commonly occur as a result of pancreatitis. Non-pancreatic RPC, on the other hand, is infrequent and has an unknown etiology. It can originate from mesentery, mesocolon or omentum [5].

Clinical presentation & investigations

There are no pathognomonic signs and characteristic symptoms for non-pancreatic RPC. Most of them attain large size before becoming symptomatic usually by compressing on adjacent structures. Patients usually present with vague abdominal pain, distension, referred pain to legs, lower limb edema, weight loss, and fever. Occasionally, patients have a sudden onset of abdominal pain which occurs due to infection or hemorrhage within the non-pancreatic RPC itself [5,6].

CT scan is a valuable tool in the evaluation of abdominal masses. It may not be able to completely differentiate benign from malignant lesions. However, it may give a clue about the diagnosis. That is why a pathological diagnosis is necessary. On CT, these lesions appear as a unilocular or multilocular cystic mass with thick walls. Occasionally, calcification of the pseudocyst wall results in an “eggshell” appearance. The contents of the pseudocyst can be hemorrhagic, purulent or serous which unlike pancreatitis is not associated with high amylase and lipase [4-7]. In our case, the patient had pain mainly in the right iliac fossa with a very low clinical suspicion of pancreatitis. Moreover, the amylase and lipase from the serum preoperatively and from the cyst fluid postoperatively were normal.

Treatment

The treatment of RPC is surgery as there is a risk of infection, rupture and malignant change. Surgical options include marsupialization, drainage and cyst excision [6]. Due to the risk of recurrence associated with residual cyst wall, complete excision is recommended [5,8]. Surgical excision can be performed trans-abdominally or retroperitoneally [6,7].

A laparoscopic approach is associated with less post-op pain, early recovery and shorter hospital stay. Pneumoperitoneum creates a space to work and with the addition of high definition angulated cameras, laparoscopy provides excellent exposure which is particularly helpful in obese patients and in the male pelvis. It also helps to identify correct tissue planes easily thus preventing inadvertent injury to adjacent vital structures [9]. In our patient, excision of the mass was challenging as it was close to the right ureter and right iliac vessels. However, by using principles of safe laparoscopic surgery it was possible to excise it completely without any intraoperative or postoperative complication (Video 1).

## Conclusions

An idiopathic non-pancreatic RPC is a rare surgical entity which carries a wide range of differential diagnosis. Surgical excision is the only way to establish a definitive diagnosis and to exclude malignancy. A laparoscopic excision should be considered if expertise is available as it is quite useful in working in narrow space. However, care should be taken to prevent cyst rupture intraperitoneally and injuring the adjacent structures.
